# Improved lumbar infusion test analysis for normal pressure hydrocephalus diagnosis

**DOI:** 10.1002/brb3.1125

**Published:** 2018-09-27

**Authors:** Erik Ryding, Babar Kahlon, Peter Reinstrup

**Affiliations:** ^1^ Department of Clinical Neurophysiology Skane University Hospital Lund Sweden; ^2^ Department of Neurosurgery King Feisal Hospital Riad Saudi Arabia; ^3^ Department of Neurosurgery Skane University Hospital Lund Sweden

**Keywords:** lumbar infusion test, normal pressure hydrocephalus, post‐operative outcome

## Abstract

**Objectives:**

Constant infusion lumbar infusion test (LIT) is an important way to find which patients, of those with signs and symptoms corresponding to normal pressure hydrocephalus (NPH) who will improve from shunt operation. LIT is a stress test on the ability for cerebrospinal fluid re‐absorbtion. The aim of this study is to show how the information from LIT can be improved by quantitative analysis and avoidance of methodological pitfalls.

**Material and methods:**

The potential pitfalls, and the analysis method, are described in detail. The analysis was applied on pre‐operative constant infusion LIT from 31 patients operated for NPH, with known outcome. The pre‐ and post‐operative walking speed was used to grade pathology progression or improvement.

**Results:**

The maximal, plateau, intra‐spinal pressure at constant infusion LIT is an ambivalent indicator for NPH: while low maximal pressure indicates no cerebrospinal fluid (CSF) absorbtion pathology, too high pressure (≥47 mmHg) may mean no diagnosis, because of stenosis of the Sylvian aqueduct. When subjects with too high intra‐spinal pressure were excluded, the new analysis gave a couple of diagnostic volume parameters, of which one appears to be an optimal LIT parameter for identifying NPH patients with 14% better accuracy than plateau pressure.

**Conclusion:**

By avoiding methodological pitfalls, and optimal analysis of the results from lumbar infusion test, the number of NPH patients who do not have a successful outcome after shunt operation may be further decreased.

## 1 INTRODUCTION

Normal pressure hydrocephalus (NPH) is a clinical condition with enlarged intra‐cerebral ventricles (Hakim & Adams, 1965), and symptoms of gait disturbance, enuresis and cognitive reduction (Fisher, 1982; Williams & Malm, 2016). The small step, shuffling gait is an early dominant symptom, which may be due to direct pressure on the midbrain gait center from an enlarged third ventricle (Lee, Yong, Ahn, & Huh, 2005). The gait disturbance offers an opportunity to evaluate the degree of disease progression (Chivukula et al., 2015; Williams et al., 2008), and may even help in prediction of good post‐operative outcome (Gaff‐Radford & Godersky, 1986).

When the underlying pathology is insufficient re‐absorbtion of CSF surgical shunting of CSF to intravenous or peritoneal space may alleviate the symptoms, but the post‐operative success depends on correct diagnosis. NPH coexists with, can be caused by, and may be mimicked by different forms of arteriosclerosis.

A direct diagnostic method for NPH is the constant infusion lumbar infusion test (LIT; Katzman & Hussey, 1970), where mock CSF is injected into the spinal cavity for passage through the Sylvian aqueduct intra‐cranially in order to stress the CSF re‐absorbtion ability.

In constant infusion LIT, the individual maximal intra‐spinal pressure at plateau level, or maximal CSF absorbtion resistance, is used as diagnostic for NPH. The LIT is theoretically simple and straightforward, but contains more information than the maximal flow resistance, Rout, and also measurement errors/pitfalls that need to be considered.

The aim of this article is to illustrate a new method to analyze LIT results, and to show its diagnostic ability by applying the analysis to LIT from a population operated for NPH, with known outcome.

## 2 MATERIALS AND METHODS

### 2.1 Subjects

The involved patient population was investigated at the Department of Neurosurgery in Lund University Hospital for possible NPH, and has been published by Kahlon, Sundbärg, & Rehncrona (2002). The patients all had dilated cerebral ventricles, small step gait disturbance, and signs of general arteriosclerosis, but only about 60% of them suffered from enuresis. Out of these subjects, 42 (21 male, 21 female) were selected for LIT with constant infusion rate.

Based on LIT and tap‐test (Wikkelsö, Anderson, Blomstrand, Lindqvist, & Svendsen, 1986) results, 31 (14 male, 17 female) subjects were selected for surgery. Their median age was 76 years, range 44‐84 years. Of these subjects, 27 had at LIT a maximal amplitude (baseline + half pulse amplitude) at or exceeding 22 mmHg (Hussey, Schanzer, & Katzman, 1970). Of the remaining four operated subjects, two had maximal amplitude of 20–21 mmHg, and two with 17–18 mmHg were selected on their results at tap‐test (improvement on cognition and walk speed, respectively).

They were operated with a ventriculo‐peritoneal or ventriculo‐venous shunt (adjustable shunt system, Codman‐Hakim model 82‐3100; Johnson & Johnson Co., Raynham, MA, USA).

This register search was accepted by the Ethical Committee of Lund University nr LU‐2014/403.

### 2.2 Walk test

Each LIT subject performed the walk test at the first (pre‐operative) test occasion, and at the second (post‐operative) median 4, 25 months later (2–22 months, except one subject 10 years, later).

During the test, the subject is instructed to walk at maximum speed for 18 meters, and mean velocity is calculated.

The difference between second and first walk speed in percent of the maximum speed (before or after) is denoted as the individual improvement, which can range from −100% to +100%.

**Figure 1:**
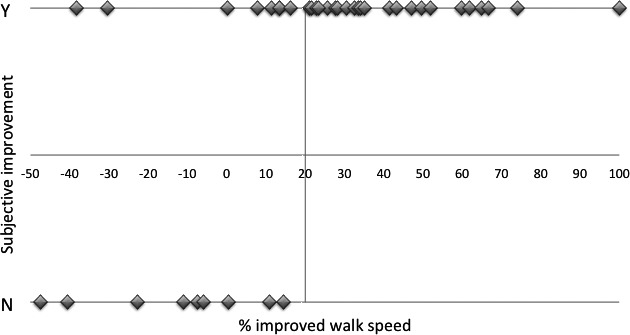
Comparison between evaluations of improvement in the lumbar infusion test subjects. Subjective feeling of improvement, yes (Y) or no (N), is related to percent walk speed increase

All the LIT subjects were asked about subjective improvement at the second examination. Reports of no subjective improvement were found at walk speed improvements up to 14% (Figure 1). Since barely perceptive improvement is not compatible with good surgical outcome, we have chosen 20% of walk speed improvement as the minimal level of good surgical outcome.

### 2.3 Lumbar infusion test

The intent for LIT is that the lumbar infusion of mock CSF will increase the intra‐cranial CSF volume. If the intra‐cranial CSF volume increases, the only volume that can decrease in equal measure is the venous volume, since the intra‐cranial tissues are incompressible. Likewise, the arterial blood volume delivered intra‐cranially during each systole is compensated for by compression of the venous pool by the same amount. The increased venous outflow resistance due to the venous vascular compression causes an increase in intra‐cranial pressure (ICP; Marmarou, Schulman, & Rosende, 1978).

The patient is lying horizontally on the side with head support. The infusion of mock CSF (Ringer solution: NaCl 8.6 g/L, KCl 0.3 g/L, CaCl 0.33 g/L; 290 mosm/kg) is through a lumbar cannula inserted into CSF space. The intra‐spinal CSF pressure is measured through another lumbar cannula. During LIT infusion, there are spino‐ventricular pressure gradients wherever there is a flow resistance. Directly following the infusion start, the spinal compliance is filled, usually causing little intra‐spinal pressure increase since the infusion is directly into the spinal cavity. Since the spinal compliance is surrounded by the in‐elastic dura mater, it does not continue to increase in volume at higher pressures. When the intra‐spinal pressure exceeds ICP, the new CSF starts entering the brain through the Sylvian aqueduct. If the perfusion pressure over the Sylvian aqueduct during LIT is neglect‐able compared to ICP then the measured intra‐spinal CSF pressure reflects ICP.

The infusion time and measured intra‐spinal pressures in this study were recorded on a calibrated printer, and these read‐outs were used as data in this study.

### 2.4 Analysis of lumbar infusion test data

**Figure 2:**
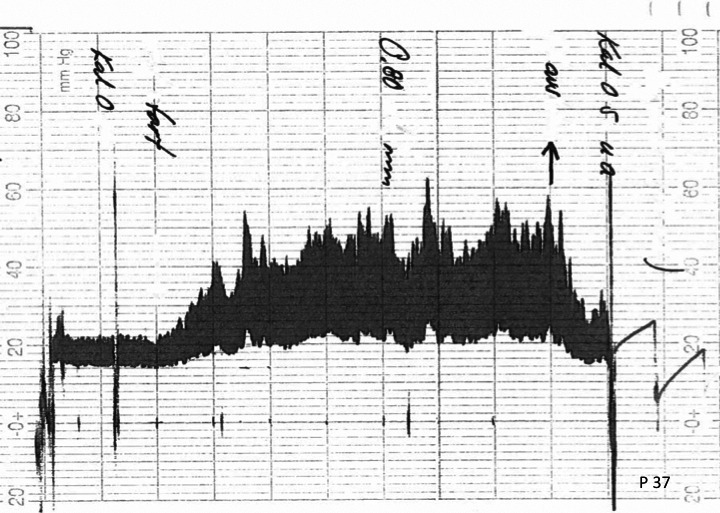
Recording of intra‐cranial pressure during a lumbar infusion test measurement in a normal pressure hydrocephalus subject. Note the exponential return to resting level when the infusion is discontinued (arrow upwards)

A typical printout of the pressure results during LIT is shown in Figure 2. The CSF infusion was at the rate 0.8 ml/min for about 20 min. Before the infusion start, there is a stable baseline superimposed by transient pressure increases (“pulse waves”) caused by arterial blood volume entering the intra‐cranial and spinal volumes at each pulse stroke. It is the basal turning points of these waves that define the basal pressure amplitude. When the intra‐spinal compliance is full, the intra‐spinal pressure increases more steeply and, when exceeding ICP, forces CSF intra‐cranially through the Sylvian aqueduct. Concomitant to the increased baseline pressure the “pulse waves” increase in amplitude. Since the CSF absorbtion increases in relation to the ICP increase, the baseline pressure increase at continued infusion gradually lessens until the pressure approaches a plateau value, *ICP*(*p*), which coincides with the maximal outflow resistance, *R*_out_ = *ICP*(*p*)/constant infusion rate. If the baseline pressure exceeds 50 mmHg, the infusion is interrupted. After interrupted infusion, the pressure exponentially decreases toward the starting value.

For analysis of LIT, knowledge of the intra‐cranial volume to ICP relationship is necessary. The Marmarou model for the intra‐cranial pressure to volume relationship (Marmarou et al., 1978) is based on a measurable intra‐cranial compliance, explained by the theory of an elastic compressible brain that, like a loaded spring, gives a mono‐exponential relation between ICP and brain volume (Avezaat, van Eijndhoven, & Wyper, 1979). However, it is a physical fact that brain tissue (including CSF) is not compressible. Since skull and dura mater forms a rigid enclosure round the brain, the only tissue that can escape at brain compression is the venous blood—but that also causes compression of the voidable venous volume, and increased outflow resistance, making an ICP increase necessary for CBF to be preserved. A detailed description is found in (Ryding, 2017).

The resulting relationship between intra‐cranial pressure and volume is according to (Ryding, 2017) given by:

(1) $$ICP=ICP\left(p\right)\times \exp\left[Vin/(Vv(p)-Vin)\right],$$

where *ICP*(*p*) is the plateau baseline ICP value, *Vin* is the increase in intra‐cranial CSF volume, and *Vv*(*p*) is the volume at plateau level of the part of the intra‐cranial venous blood volume that can be decreased due to infusion of CSF. The ICP reference level (mean central venous blood pressure at spinal and mid‐cerebral level) is set to zero. For easier computation, *ICP*(*p*) was chosen as infusion reference point, which makes *Vin* negative, reaching zero at plateau level.

If Equation (1) is re‐written as:

(2) $$Vin=Vv\left(p\right)\times \frac{\ln\left(\frac{\mathrm{ICP}}{ICP(p)}\right)}{1+\ln\left(\frac{\mathrm{ICP}}{ICP(p)}\right)}$$

It is obvious that it describes the relationship between *Vin* and *Vv*(*p*). Since *Vv*(*p*) does not change during the infusion, it is a reference point for the *Vin* change. At the start of the intra‐cranial infusion, *Vin* is:—maximal increase in intra‐cranial CSF volume, −*Vin*(*max*), and Equation (2) gives its relation to *Vv*(*p*).

**Figure 3:**
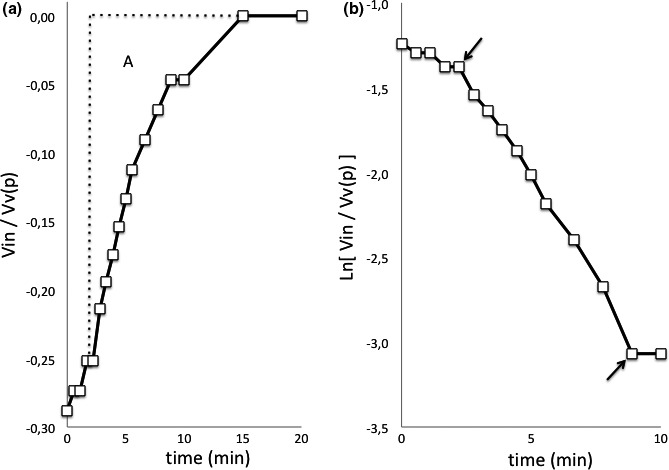
(a) Increase in the intra‐cranial cerebrospinal fluid (CSF) volume (*Vin*) in relation to reference voidable venous volume at plateau pressure (*Vv*(*p*)), calculated from basal intra‐cranial pressure values in Figure 2, which gives exponential approach to the *Vv*(*p*) level. The area, *A*, between *Vv*(*p*) and the exponential approach gives the infusion time for the CSF volume increase. (b) Logarithmic plot of the distance from the *Vv*(*p*) level in (a) with the linear part between arrows giving the exponential, *K*, and *A* = 1/*K* = 4.3 min, and *Vin* = 4.3 × 0.8 ml. The slope before the first arrow illustrates the filling of the intra‐spinal compliance

Figure 3a illustrates the *Vin* corresponding to the basal LIT pressure curve in Figure 2. The shape of the *Vin* curve indicates that it starts at the intra‐cranial infusion start with −*Vin*(*max*) and increases by exponentially approaching 0 at *Vv*(*p*). The area between *Vv*(*p*) and the *Vin* curve from intra‐cranial infusion start gives the effective infusion time at the given infusion rate for the part of the CSF infusion remaining intra‐cranially.

Since the *Vin* curve approaches exponentially the *Vv*(*p*) level, logarithmation of the distance between the *Vin* curve and the *Vv*(*p*) level gives a straight line with a slope corresponding to the exponential, *K* (per minute), which is shown in Figure 3b. In order to minimize movement errors, the slope is best measured between its end‐points.

Since the area, A, under the exponential curve is 1/*K*, the exponential gives the effective infusion time, and with the infusion rate 0.8 ml/min also the part of the infusion volume remaining intra‐cranially, *Vin*(*max*). By using the relationship between *Vin*(*max*) and *Vv*(*p*) at the intra‐cranial infusion start, *Vv*(*p*) can be calculated.

*Vv* at the time of beginning of CSF inflow, *Vv*(0), is then known since:

(3) Vv(0)=Vv(p)+Vin(max).

### 2.5 Resting “pulse volume”

The increase in ICP at rest, before infusion, from intra‐cranial infusion of an arterial blood volume at each pulse stroke enables calculation of the maximal infused blood volume, *Vpulse*. Equation (2) gives:

(4) $$Vpulse=Vv\left(0\right)\times \frac{\ln\left(\frac{ICP(0)}{ICP(pluse)}\right)}{1+\ln\left(\frac{ICP(0)}{ICP(pulse)}\right)},$$

where *ICP*(0) is basal ICP before infusion, and *ICP*(*pulse*) is the maximal ICP of the “pulse waves” above *ICP*(0).

### 2.6 Statistics

Statistical evaluation of the ability of the result parameters to differentiate between subjects with at least 20% post‐operative walk speed increase (successful outcome), and those with less successful outcome, was made with the Kolmogorov–Smirnov non‐parametric test.

## 3 RESULTS

### 3.1 Pre‐operative walk speed

**Figure 4:**
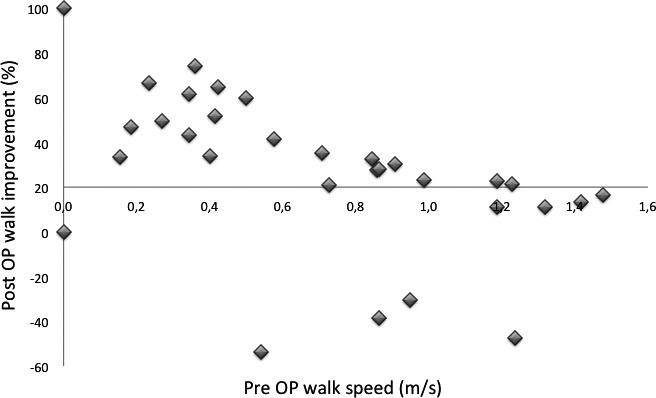
Initial walk speed compared to post‐operative percent walk speed increase gave a *p* = 0.02 separation between successful (20% walk speed increase or more) and less successful post‐operative results. Absent walking ability did not give any outcome indication. In the normal pressure hydrocephalus patients, higher initial walking speed tended to indicate lower level of post‐operative improvement

Pre‐operative walk speed showed a *p* = 0.02 ability to identify subjects who had at least 20% post‐operative improved walk speed from those who did not. If the patient can walk, a walk speed below 1 m/s before operation indicated an increased probability for at least 20% increased post‐operative walk speed (Figure 4). Four subjects break the pattern; all with substantial worsening of the post‐operative walk speed. Of these four, one had maximal LIT amplitude of 17 mmHg, and two had LIT amplitudes of 45 mmHg or more.

### 3.2 LIT plateau pressure (baseline plus half pulse‐height)

**Figure 5:**
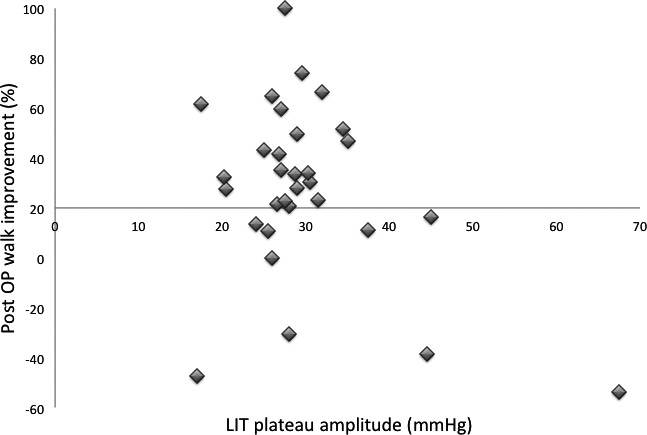
LIT plateau pressure in relation to post‐operative 20% increase in walk speed gave no significant separation between the successful/less successful post‐operative groups. Of the four subjects with plateau pressure above 37 mmHg, all had less than 20% post‐operative walk speed improvement, like the subject with plateau pressure 17 mmHg

LIT plateau pressure did not show any significant ability to identify subjects who had at least 20% post‐operative improved walk speed from those who did not. 28 subjects had plateau pressure above 21 mmHg, and of these 8 (29%) had <20% increased post‐operative walk speed. Subjects with at least 20% increased post‐operative walk speed had plateau pressure in the range 18–35 mmHg. In Figure 5, all four subjects with plateau pressure 38–68 mmHg had <20% increased post‐operative walk speed.

With the four subjects with plateau pressure at or above 38 mmHg removed, 4 subjects (14%) with plateau pressure above 21 mmHg had <20% increased post‐operative walk speed.

### 3.3 Diagnostic volume parameters

**Figure 6:**
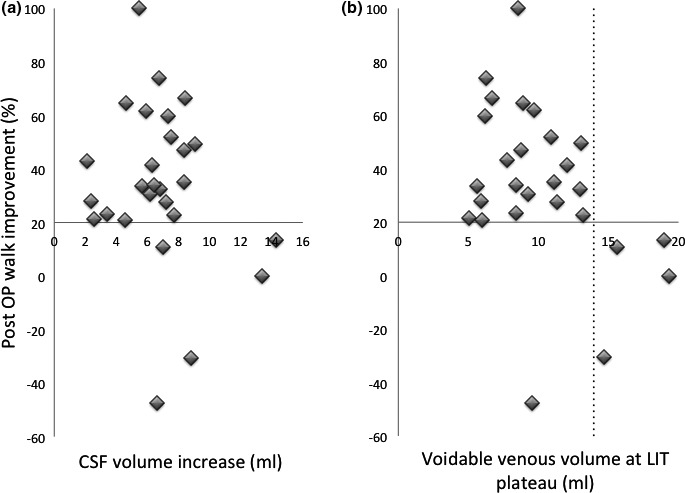
New LIT parameters for pre‐operative NPH diagnosis. (a) The intra‐cranial increase in CSF volume, *Vin*, during infusion gave no significant separation between successful (20% walk speed increase or more) and less successful post‐operative results. (b) The intra‐cranial volume of voidable venous blood at plateau level, *Vv*(*p*) gave a *p* = 0.01 separation between successful (20% walk speed increase or more) and less successful post‐operative results. The one subject with *Vv*(*p*) below 14 ml and less successful post‐operative result had 17 mmHg LIT plateau pressure, and was on that ground no NPH subject

The subjects with plateau pressure >37 mmHg may have erroneously high intra‐spinal pressure due to high resistance over the Sylvian aqueduct, and consequently erroneous calculated volume results. With the results from these subjects removed, the *Vin*(*max*) and *Vv*(*p*) for the operated subjects are shown in Figure 6.

The *Vin*(*max*) and *Vv*(*p*) were evaluated for their diagnostic ability to differentiate between subjects with 20% or more increased post‐operative walk speed or not. For both parameters, a lower volume indicates a higher probability of NPH.

*Vin*(*max*) did not show any significant ability to identify subjects who had at least 20% post‐operative improved walk speed from those who did not, although subjects with *Vin*(*max*) less than 7 ml all but one had 20% or more increased post‐operative walk speed. The one subject with decreased post‐operative walking ability despite *Vin*(*max*) <7 ml was the one with the low, LIT plateau pressure of 17 mmHg.

Almost all subjects with *Vv*(*p*) less than 14 ml had had 20% or more increased post‐operative walk speed, which gave a *p* = 0.01 ability to identify subjects who had at least 20% post‐operative improved walk speed from those who did not. The one subject who breaks this pattern with decreased post‐operative walking ability despite *Vv*(*p*) 10 ml, was the one with the low LIT plateau pressure of 17 mmHg (and no walk speed improvement on tap‐test).

### 3.4 Baseline ICP, Vv(0), and “pulse wave” volume at rest

For subjects with ICP plateau pressure at LIT of 18–30 mmHg (*n* = 22), all NPH patients, the baseline ICP was, mean: 9.3 range: 6–14 mmHg, *Vv*(0) was mean: 15 range: 8–22 ml, and “pulse wave” volume was mean: 5.2 range: 3–7 ml.

For subjects with ICP plateau pressure at LIT less than 18 mmHg (*n* = 6), not NPH patients but with cerebral subcortical arteriosclerosis, the baseline ICP was, mean: 6.4 range: 5–8 mmHg, *Vv*(0) was mean: 19 range: 13–30 ml, and “pulse wave” volume at rest was mean: 5.6 range: 4–10 ml.

## 4 DISCUSSION

The aim of this study was to find an optimal analysis of lumbar infusion test, with constant infusion rate, for diagnosis of NPH. The tool for evaluation of diagnostic success has been LIT results from 31 patients operated for NPH, with known outcome. Diagnostic criteria during constant flow infusion have been the maximal intra‐spinal pressure, plateau pressure, during infusion (Hussey et al., 1970) or the corresponding CSF outflow resistance (Boon et al., 1998) that at constant infusion rate contains identical information.

The first encountered methodological difficulty was that ICP during CSF infusion is measured in the spinal cavity. It is then essential that the CSF flow resistance over the Sylvian aqueduct is neglectable compared to the intra‐cranial CSF outflow resistance, since if increased it invalidates the LIT results. Magnetic resonance measurement of pulse synchronous CSF flow over the Sylvian aqueduct in NPH patients indicates that increased flow resistance over the Sylvian aqueduct gives no diagnostic information on NPH (Kahlon, Annertz, Ståhlberg, & Rehncrona, 2007). The four subjects in this study with plateau pressure above 37 mmHg appear to belong to this group since they had widely variable post‐operative outcome, but in no case 20% or more increased post‐operative walk speed. In consequence to our findings, it appears that selection for surgery based on LIT plateau pressure cannot be based simply on amplitude more than 21 mmHg (Hussey et al., 1970) but also not above 37 mmHg, where the group with plateau pressure above 37 mmHg should be selected for surgery by additional testing (Marmarou, Bergsneider, Klinge, Relkin, & Black, 2005). In fact, subjects with plateau pressure less than 22 mmHg may have NPH, since three subjects with plateau pressure 18–21 mmHg had 20% or more increased post‐operative walk speed, although one with plateau pressure 17 mmHg did not improve.

The new, improved analysis of the LIT results was intended to give a test parameter with improved diagnostic accuracy. Obvious candidates for diagnostic parameters were the maximal increase in CSF volume intra‐cranially during the infusion, *Vin*(*max*), and the intra‐cranially remaining part, *Vv*(*p*), of the venous blood volume that leaves intra‐cranial space as compensation for *Vin*(*max*) entering.

It is evident from Figure 5 that *Vv*(*p*) appears to be the better diagnostic parameter, with total separation between subjects who had 20% or more increase in post‐operative walking speed, and those who did not, except for one subject with the low LIT plateau pressure of 17 mmHg, and no walk speed improvement on tap‐test. In comparison, of the subjects with 21–35 mmHg plateau pressure 14% had less than 20% walk speed improvement. Additionally, the *Vv*(0) parameter, which is the voidable part of the intra‐cranial venous volume before infusion start, is of importance for cerebral blood flow, CBF, since it indicates the maximal limit for the arterial blood volume that can enter intra‐cranially at each heartbeat, and a limitation of CBF is coupled to the cognitive decline at advanced NPH.

The measurement results for basal ICP, “pulse volume” before infusion start, and *Vv*(0) were not useful parameters for NPH diagnosis.

Theoretically, an optimal measurement situation for LIT includes an intra‐cranial ICP measurement, and measurement of mean central venous pressure, CVP, with sensors at the same horizontal level. Intra‐cranial ICP, measured with mean CVP as zero level, at LIT would avoid errors due to Sylvian aqueduct stenosis and minimize those of coughing and tilting during recording (described below). However, such a “gold standard” recording is somewhat more invasive than the basic setup used in this study.

Sources of error in LIT results, besides an increased CSF flow resistance over the Sylvian aqueduct, are errors in the reference level to the intra‐spinal pressure measurements. The patient lies on one side, and if he leans forward, or backward, the central venous pressure decreases, and consequently pressure in cervical veins, giving a recorded ICP decrease unrelated to the intra‐cranial CSF infusion. Coughs, grunts, and head lift increase the intra‐thoracic pressure, the pressure in cervical veins, and ICP. If the body position is not horizontal from the lumbar to the cervical region, there will be a bias in the pressure measurements, which will affect the relationship between the *Vin*(*max*) and *Vv*(*p*).

Providing a stable, comfortable, easily adjustable recording site, with physical and mental support, will reduce most of these errors.

Errors of these types exist in varying degree in the LIT measurements used to validate the new analysis method. The limited number of subjects, 31, also affects the quality of the analysis results. As a consequence, the here presented analysis results should be regarded as a first estimate, later to be confirmed, rather than final limits.

In conclusion, when constant infusion LIT is used for diagnosis of NPH, avoidance of the methodological errors/pitfalls, and the improved analysis described here, may substantially improve diagnosis, and consequently post operative outcome.

## CONFLICT OF INTEREST

The authors have no conflict of interests to disclose.
